# Clinical evaluation of ultrasonic subgingival debridement versus ultrasonic subgingival scaling combined with manual root planing in the treatment of periodontitis: study protocol for a randomized controlled trial

**DOI:** 10.1186/s13063-019-4031-y

**Published:** 2020-01-28

**Authors:** Yue Yan, Yalin Zhan, Xian’e Wang, Jianxia Hou

**Affiliations:** 1 Department of Periodontology, Peking University School and Hospital of Stomatology and National Engineering Laboratory for Digital and Material Technology of Stomatology and Beijing Key Laboratory of Digital Stomatology, 22 Zhongguancun South Avenue, Haidian District, Beijing, 100081 People’s Republic of China; 2 Department of General Dentistry, First Clinical Division, Peking University School and Hospital of Stomatology and National Engineering Laboratory for Digital and Material Technology of Stomatology and Beijing Key Laboratory of Digital Stomatology, Beijing, China

**Keywords:** Periodontitis, Non-surgical periodontal therapy, Ultrasonic subgingival debridement, Root planing

## Abstract

**Background:**

Periodontal diseases are regarded as the most common diseases of mankind. The prevalence rate of periodontal disease assumes a clear growth tendency, increasing by 57.3% from 1990 to 2010. Thereby, effective periodontal therapy is still a long-term task and a difficult problem. The goals of periodontal therapy are to eliminate the infectious and inflammatory processes of periodontal diseases. Root planing, in order to eliminate the “infected cementum,” has been an important step in the treatment of periodontitis since the 1970s. However, along with the understanding of the effects of endotoxin on the root surface, the necessity of manual root planing has been gradually queried. Ultrasonic instruments, which are more recent innovations, would not remove the cementum excessively, and are also more time-saving and labor-saving compared to using hand instruments. Hence, an increasing number of dentists prefer to do scaling with ultrasonic instruments only. However, the necessity of root planing remains emphasized in the international mainstream views of periodontal mechanical treatment. Therefore, this study is devoted to compare the clinical effect of ultrasonic subgingival debridement and ultrasonic subgingival scaling combined with manual root planing, which takes the implementation of root planing as the only variable and is more in line with the current clinical situation, thus hoping to provide some valuable reference to dentists.

**Methods/design:**

Forty adult patients who fit the inclusion criteria are being recruited from the Peking University Hospital of Stomatology (Beijing, China). By means of randomization tables, one quadrant of the upper and lower teeth is the test group and the other is the control group. Test group: ultrasonic subgingival scaling combined with manual root planing. Control group: ultrasonic subgingival debridement. In a 24-week follow-up period, plaque index, probing depth, clinical attachment loss, bleeding index, furcation involvement, mobility, and patient-reported outcome (Visual Analog Scale for pain and sensitivity) will be observed and documented.

**Discussion:**

This study evaluates the effectiveness of ultrasonic subgingival scaling combined with manual root planing and ultrasonic subgingival debridement alone in the nonsurgical treatment of periodontitis with a split-mouth design after 1, 3 and 6 months. The result of the trial should potentially contribute to an advanced treatment strategy for periodontitis with an ideal clinical outcome.

**Trial registration:**

International Clinical Trials Registry Platform (ICTRP), ID: ChiCTR1800017122. Registered on 12 July 2018.

## Background

Periodontal diseases are regarded as the most common diseases of mankind [1]. The prevalence rate of periodontal disease assumes a clear growth tendency worldwide, increasing by 57.3% from 1990 to 2010 [2–5]. The Global Burden of Disease Study [6] reports that periodontitis is the sixth most prevalent disease worldwide. The overall prevalence is 11.2% or around 743 million people in the worldwide. The prevalence of periodontitis in China is even higher. According to the recently released Fourth Epidemiologic Sampling Survey in China, the prevalence of periodontal disease is 90.9% in the age group of 35–44 years. Periodontitis is the main cause of tooth loss in the adult population worldwide that affects nutrition, quality of life and self-esteem as well as imposing great socio-economic impacts and healthcare costs [7–10]. However, good periodontal therapy is still a long-term task and a difficult problem. Periodontal treatment aims to control gingivitis and periodontitis, avoid disease progression leading to tooth loss, retain a functional dentition for a lifetime, preserve self-esteem and improve quality of life.

Subgingival plaque and calculus on the root surface in the periodontal pocket are the most important local factors for the occurrence and development of periodontitis. Hence, the ultimate goal of nonsurgical pocket/root instrumentation is to render the root free from microbial deposits and calculus. In the past, dentists used a variety of manual instruments (e.g., scalers, curettes) to remove these local irritants. Later, it was found that the vibration of ultrasonic tips, as well as the cavitation effect and microflow of cooling water could effectively remove plaque and calculus, which has made ultrasonic instruments widely used in nonsurgical treatment of periodontitis. These methods are collectively referred to as “subgingival scaling.” In the 1970s, Hatfield and Aleo found that endotoxin could penetrate to the cementum and influence the attachment of fibroblasts, leading to the concept of “root planing,” which means that the root surface should be smoothed by manual instruments to effectively remove the infected cementum, so as to remove endotoxin and form a smooth, hard and clean root surface with biocompatibility, which is conducive to the attachment and healing of periodontal tissue [11–13]. By the 1980s, more studies clarified that endotoxin was only loosely attached to the surface of the cementum [14, 15], and most of the endotoxin was related to the bacterial biofilm [16–18]. Therefore, it was suggested that excessive root planing for endotoxin removal was unreasonable. In 1994, the first European Working Conference on periodontology reached a consensus on the term “subgingival debridement,” that is, to use a gentle method to remove subgingival plaque and calculus, and to preserve the cementum as far as possible [19]. Ultrasonic instruments will not remove the cementum excessively, and are also more time-saving and labor-saving compared to hand instruments. Hence, an increasing number of dentists prefer to do scaling with ultrasonic instruments only. However, the necessity of root planing remains emphasized in the international mainstream views of periodontal mechanical treatment; ultrasonic subgingival scaling with manual root planing is recommended after supragingival scaling.

The primary objective of scaling and root planing is to restore gingival health by completely removing elements that provoke gingival inflammation (i.e., biofilm, calculus and endotoxin) from the tooth surface. A large amount of in vivo and in vitro research has been conducted to compare ultrasonic and manual instruments, and it was found that there was not much difference between them in clinical effects, changes in microflora and root surface characteristics [20–25]. In fact, there is still a lack of research evidence comparing the clinical effects of ultrasonic subgingival scaling with or without manual root planing. On the one hand, it is difficult to distinguish subgingival scaling from root planing in traditional manual operation; on the other hand, there are fewer dentists using manual subgingival scaling and root planing. Therefore, this study is devoted to comparing the clinical effect of ultrasonic subgingival debridement and ultrasonic subgingival scaling combined with manual root planing in the nonsurgical treatment of periodontitis, which, taking the implementation of root planing as the only variable, is more in line with the clinical situation, hoping to provide some valuable reference for dentists.

### Objectives and hypotheses

The major goals of the current randomized controlled trial are to compare and evaluate the clinical outcomes of ultrasonic subgingival debridement and ultrasonic subgingival scaling combined with manual root planing.

The primary hypotheses are root planing is an important step in nonsurgical treatment of periodontitis in order to remove subgingival plaque and calculus, eliminate the “infected cementum” and promote healing.

## Methods/design

### Overview

The study is a prospective, single-center, split-mouth randomized controlled trial. Forty patients who have periodontitis and are in need of periodontal treatment will be recruited. The assessments, interventions and follow-ups will be performed at Peking University School and Hospital of Stomatology (Beijing, China). This study has been approved by the Ethics Committee of Peking University School and Hospital of Stomatology (PKUSSIRB-201734032) and registered in the International Clinical Trials Registry Platform (ICTRP) under the ID: ChiCTR1800017122.

### Inclusion criteria

Aggressive periodontitis (AgP): according to the AgP diagnostic criteria established by the international symposium on the classification of periodontal diseases in 1999 [26]:

1. Aged 18 to 35 years
2. Rapid alveolar bone destruction and attachment loss: the probing depth of at least six teeth (at least three of them are non-first molars and incisors) in the whole mouth is greater than 5 mm, and the adjacent attachment loss is greater than 3 mm. All are confirmed to have alveolar bone resorption on the adjacent surface by examining periapical films
3. There are at least 20 remaining teeth in the whole mouth except the third molars, and at least one molar in each quadrant

Chronic periodontitis (CP): according to the CP diagnostic criteria established by the International Symposium on the Classification of Periodontal Diseases in 1999 specifies that [26]:

1. The patient’s onset age is between 35 and 60 years
2. The patient is systematically healthy, has gingival bleeding, swelling, pain, halitosis, teeth mobility and occlusive discomfort
3. At least one molar exists in each quadrant. At least two sites in each quadrant with a probing depth greater than 5 mm have attachment loss greater than 3 mm
4. At least 50% of all teeth in the whole mouth have the following conditions: (a) there exists sites with a probing depth greater than 5 mm; (b) alveolar bone absorption is greater than or equal to 30%; (c) there is bleeding on probing or periodontal abscess
5. There are at least 20 remaining teeth in the whole mouth except the third molars

### Exclusion criteria

1. Patients have received periodontal treatment within 6 months or taken antibacterial drugs within 3 months
2. Pregnant women or women of child-bearing age who do not take effective contraceptive measures
3. Systemic diseases such as cardiovascular and endocrine disease are present
4. Allergic to penicillin
5. Smokers and those who cannot give up alcohol while taking drugs
6. Patients do not agree to participate in the trial, and do not sign the informed consent

### Recruitment

**Fig. 1:**
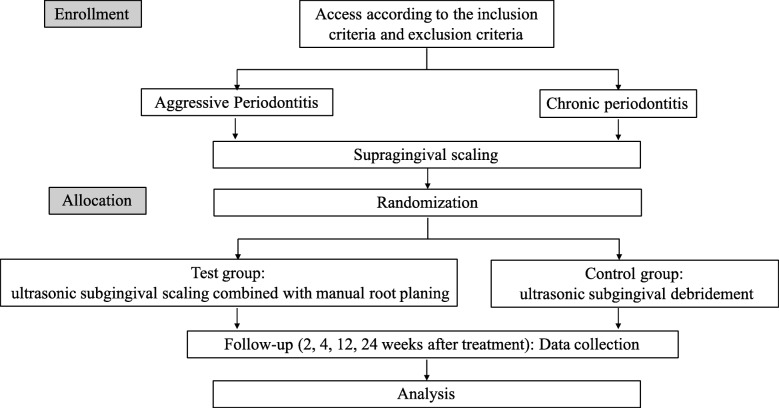
Consolidated Standards of Reporting Trials (CONSORT) diagram

Subjects who are looking for periodontal treatment and are willing to join this trial will be recruited from the Periodontology Department, Peking University School and Hospital of Stomatology. Subjects will receive the study information. Before subjects are included in the present study, the consent form must be signed. Figure 1 shows the flow procedure of participants through this trial.

### Groups, randomization and blinding

An experienced periodontist is going to perform the periodontitis disease diagnostic process according to clinical and radiographic examination. The randomization sequence and allocation concealment (placed in sealed envelopes) were performed by a professor in the absence of the working investigators; one quadrant of the upper and lower teeth is the test group and the other quadrant is the control group. Test group: ultrasonic subgingival scaling combined with manual root planing. Control group: ultrasonic subgingival debridement. All subjects will be treated by one experienced and calibrated therapist who does not partake in the allocation, examination, or statistical analysis. The treatment plan and grouping will be confidential to the examiner and statistical analyst.

### Interventions

All enrolled subjects received supragingival scaling using ultrasonic scalers, oral hygiene instruction (OHI) including tooth brushing with the modified Bass technique and interdental cleaning with interdental brushes or dental floss. They are submitted to a complete periodontal clinical assessment. Subsequently, the upper and lower teeth of each subject are randomly allocated to the following therapeutic groups: (1) ultrasonic subgingival scaling combined with manual root planing group; (2) ultrasonic subgingival debridement group. The treatment is carried out at the Periodontology Department, Peking University School and Hospital of Stomatology. The treatment is performed by an experienced periodontist who has been calibrated before the trial. The treatment is completed under local anesthesia in two sessions of approximately 1 h, distributed over a period of 7 days. Ultrasonic subgingival scaling is performed using magnetostrictive ultrasonic device (Charlotte, North Carolina Dentsply, model number: Gen-130B, 25 kHz, USA). Manual root planing is performed using Gracey curettes (conventional and mini-fives) numbers 5/6, 7/8, 11/12 and 13/14 (Hu-Friedy, Shanghai, China). At the end of each session, the clinical coordinator evaluates the effectiveness of treatment using the outcome “smoothness of the scaled roots.” All subjects will receive personalized OHI after treatment until the end of the study (24 weeks post therapy).

### Examination

#### Baseline examination

After the subjects have been included, plaque index (PLI), probing depth (PD), clinical attachment loss (CAL), bleeding index (BI), furcation involvement (FI) and mobility are tested before treatment by a calibrated examiner (not the therapist) who has been trained to adequate levels of accuracy and reproducibility.

### Examination during the follow-ups

#### Follow-up

All subjects will be recalled for follow-up at weeks 2, 4, 12 and 24 after the treatment. At weeks 4, 12 and 24 after the treatment, PLI, PD, CAL, BI, FI and mobility will be examined by a calibrated examiner. Any complications will be documented. Besides, subjects will complete a Visual Analog Scale/Score (VAS) to evaluate pain and sensitivity during the 2 and 4 weeks after treatment.

### Primary parameters

The primary parameters of this trial are PD, CAL and BI.

### Secondary parameters

The secondary parameters of this trial include PLI, FI, mobility and VAS to evaluate pain and sensitivity after treatment.

### Sample size

The sample size of this trial is calculated based on the formula:

$$ \kern13.25em N=\left[\frac{\left(z\frac{\alpha }{2}+ z\beta \right)\sigma }{\delta}\right]2\left(\frac{1}{Q1}+\frac{1}{Q2}\right). $$

According to the preliminary experiment results and data analysis from currently published articles, the difference of PD with and without root planing (*δ*) is around 0.3 mm and the standard deviation in groups (*σ*) is around 0.2 mm.

If the inspection level (*α*) is set at 0.05 and the power of test (*β*) is set at 90%, then 18 subjects will be required for each group. Given that loss to follow-up is around 10%, this study will require 20 subjects for each group. Consequently, this trial will require at least 40 subjects in all.

### Timeline

**Fig. 2:**
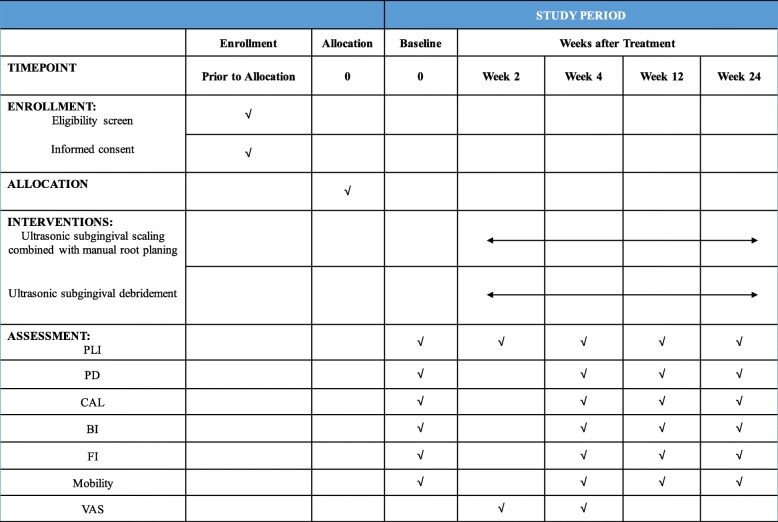
The schedule of enrollment, intervention and assessments. Abbreviations: *PLI* plaque index, *PD* probing depth, *CAL* clinical attachment loss, *BI* bleeding index, *FI* furcation involvement, *VAS* Visual Analogue Scale/Score

The recruitment began in October 2018, and the intervention period will be ending in June 2020. Figure 2 shows the schedule of enrollment, interventions and assessments.

### Data collection and management

The data of the patients will be documented on both spreadsheets and databases. The statistical analysis will be performed by two experimenters independently.

### Statistical analysis

A Shapiro-Wilk test and Levene variance homogeneity test will be performed to test the normality and variance equality, respectively. Continuous normally distributed data will be expressed as mean ± standard deviation (SD), and non-normally distributed data as median (lower to upper quartile). A paired-samples *T* test or two-related-samples test will be used to identify any differences between groups. Statistical significance difference will be set as *P* value of less than 0.05. Data analyses will be performed using SPSS software.

### Ethical considerations

#### Ethical approval

The trial has been approved by the Ethics Committee of Peking University School and Hospital of Stomatology (PKUSSIRB-201734032). Before subjects are officially recruited into this study, they will be given a study information sheet and will be asked to sign a consent form.

### Withdrawal

Subjects will be informed that they have the right to withdraw from this trial at any time without providing a reason. If the withdrawal occurs, treatment will also be provided to the subject.

### Dissemination of results

The results of this trial will be saved at the International Clinical Trials Registry Platform (ICTRP) and published in an international peer-reviewed journal which will allow anyone access to obtain the results.

## Discussion

Periodontitis is strongly associated with the presence of bacterial biofilms and dental calculus on root surfaces. Hence, the ultimate goal of nonsurgical pocket/root instrumentation is to render the root free from microbial deposits and calculus. The success of periodontal treatment depends on the removal of deposits from the root surface [27–30]. All kinds of studies performed in different models and under different conditions have indicated that neither manual nor mechanical instruments are superior in removing subgingival deposits [31–37]. There was no significant difference in the changes of PD, CAL and BOP between manual subgingival debridement and ultrasonic subgingival debridement.

Previous studies demonstrated that hand instrumentation curettage created the smoothest root surface, whereas mechanical instruments, such as the ultrasonic scaler, tended to roughen the root surface [38]. Cobb found that manual curettes were more technique-sensitive and time-consuming [39]. The old concept of infected cementum removal in order to provide the root surface biocompatible for soft tissue healing [11, 12] has been questioned by various studies [15, 16]. The utilization of ultrasonic devices for subgingival debridement offers a less aggressive and a more comfortable therapeutic method for both the patient and therapist. But, some research has shown that the comparison between manual instruments and ultrasonic scalers did not show an advantage over machine-driven instruments [20], and tissue trauma was similar in both instruments [40]. Therefore, the necessity of manual root planing cannot be completely denied. Hand instrumentation has been recommended to smooth the root surface after ultrasonic debridement as the final finishing procedure in the treatment of periodontitis-affected roots [41]. At present, root planing is no longer used to emphasize the deliberate removal of cementum, but to contribute to the removal of subgingival plaque.

This study thus intends to evaluate in vivo the effectiveness of ultrasonic subgingival scaling combined with manual root planing and ultrasonic subgingival debridement alone in the nonsurgical treatment of periodontitis with a split-mouth design after 1, 3 and 6 months. We hope that the results could lead to an advanced treatment strategy of periodontitis with an ideal clinical outcome.

### Trial status

The trial has been registered at International Clinical Trials Registry Platform (ICTRP) under the identifier number ChiCTR1800017122 on 12 July 2018. The recruitment began in October 2018 and will be completed in June 2020.

## Supplementary information

**Additional file 1.** Standard Protocol Items: Recommendations for Interventional Trials (SPIRIT) 2013 Checklist: recommended items to address in a clinical trial protocol and related documents*

## Data Availability

Not applicable

## Abbreviations

AgP: Aggressive periodontitis
BI: Bleeding index
CAL: Clinical attachment loss
CP: Chronic periodontitis
FI: Furcation involvement
ICTRP: International Clinical Trials Registry Platform
PD: Probing depth
PLI: Plaque index
SD: Mean ± standard deviation
VAS: Visual Analog Scale/Score
